# Multi-Omic Data Interpretation to Repurpose Subtype Specific Drug Candidates for Breast Cancer

**DOI:** 10.3389/fgene.2019.00420

**Published:** 2019-05-07

**Authors:** Beste Turanli, Kubra Karagoz, Gholamreza Bidkhori, Raghu Sinha, Michael L. Gatza, Mathias Uhlen, Adil Mardinoglu, Kazim Yalcin Arga

**Affiliations:** ^1^Department of Bioengineering, Marmara University, Istanbul, Turkey; ^2^Science for Life Laboratory, KTH – Royal Institute of Technology, Stockholm, Sweden; ^3^Department of Bioengineering, Istanbul Medeniyet University, Istanbul, Turkey; ^4^Department of Radiation Oncology, Rutgers Cancer Institute of New Jersey, New Brunswick, NJ, United States; ^5^Department of Biochemistry and Molecular Biology, Penn State College of Medicine, Hershey, PA, United States; ^6^Faculty of Dentistry, Oral and Craniofacial Sciences, Centre for Host-Microbiome Interactions, King’s College London, London, United Kingdom; ^7^Department of Chemical and Biological Engineering, Chalmers University of Technology, Gothenburg, Sweden

**Keywords:** breast cancer, drug repositioning, non-cancer therapeutics, repurposing, basal subtype, personalized metabolic models

## Abstract

Triple-negative breast cancer (TNBC), which is largely synonymous with the basal-like molecular subtype, is the 5th leading cause of cancer deaths for women in the United States. The overall prognosis for TNBC patients remains poor given that few treatment options exist; including targeted therapies (not FDA approved), and multi-agent chemotherapy as standard-of-care treatment. TNBC like other complex diseases is governed by the perturbations of the complex interaction networks thereby elucidating the underlying molecular mechanisms of this disease in the context of network principles, which have the potential to identify targets for drug development. Here, we present an integrated “omics” approach based on the use of transcriptome and interactome data to identify dynamic/active protein-protein interaction networks (PPINs) in TNBC patients. We have identified three highly connected modules, EED, DHX9, and AURKA, which are extremely activated in TNBC tumors compared to both normal tissues and other breast cancer subtypes. Based on the functional analyses, we propose that these modules are potential drivers of proliferation and, as such, should be considered candidate molecular targets for drug development or drug repositioning in TNBC. Consistent with this argument, we repurposed steroids, anti-inflammatory agents, anti-infective agents, cardiovascular agents for patients with basal-like breast cancer. Finally, we have performed essential metabolite analysis on personalized genome-scale metabolic models and found that metabolites such as sphingosine-1-phosphate and cholesterol-sulfate have utmost importance in TNBC tumor growth.

## Introduction

Breast cancer is the most commonly diagnoses and second leading cause of cancer-related deaths in women in the United States with an estimated 268,600 new cases and 41,760 deaths in 2019 (Siegel et al., 2019). Although overall survival has significantly improved over the past several decades owing in part to advances in early diagnostic techniques and an increasing understanding of the underlying biological basis of the disease, which has led to improved treatment strategies. On a molecular level, breast cancer can be defined as five predominant molecular subtypes including the luminal A (LumA), luminal B (LumB), and Normal-like (NL) subtypes which are predominantly estrogen receptor (ER) and progesterone receptor (PR) positive; the HER2 Enriched subtype (HER2E) subtype; and basal-like tumors which are largely synonymous with Triple Negative Breast cancer (TNBC) and are ER/PR/HER2 negative. The considerable differences among these molecular subtypes are a consequence of dramatically altered genomic and proteomic profiles which manifest as changes in activated signaling networks (Gatza et al., 2014) and manifest as differences in risk factors, incidence, age, prognosis and response to treatment. Therefore, there is a clear need to develop reliable biomarkers and to identify potential drug targets in each molecular and clinical subtype (Perou et al., 2000; Curtis et al., 2012; Weigman et al., 2012; Gatza et al., 2014; Ciriello et al., 2015; Mertins et al., 2016).

Basal-like breast cancers disproportionally affect younger women and women of African American decent. This subtype, which is highly concordant with TNBC, accounts for ∼15–20% of diagnosed breast tumors but more than 1-in-4 breast cancer related deaths each year. This is, due in part, to the lack of effective therapeutic options for TNBC patients aside from multi-agent chemotherapy, which remains the standard-of-care treatment despite a limited and varied response among patients and the related toxic side-effects (Solzak et al., 2017). In this context, we and others, have proposed that systems level analyses can assist in revealing the underlying molecular mechanism of the diseases, discovery of biomarkers for specific subtypes, identification of subtype specific drug targets and reposition of drugs that can be used in effective treatment of patients (Mardinoglu and Nielsen, 2015; Mardinoglu et al., 2018; Turanli et al., 2018).

Publicly available “omics” datasets including The Cancer Genome Atlas (TCGA) (Ciriello et al., 2015), Molecular Taxonomy of Breast Cancer International Consortium (METABRIC) (Curtis et al., 2012), and the National Cancer Institute’s Clinical Proteomic Tumor Analysis Consortium (NCI-CPTAC) (Mertins et al., 2016) enhance our understanding of the subtype specific molecular mechanisms of breast cancer. Moreover, integrative and comparative analysis of “omics” data together with network modeling provided a comprehensive platform for the drug repositioning and multi-target drug design (Kibble et al., 2015; Vitali et al., 2016; Turanli et al., 2017). A number of studies also combined genomic, transcriptomic, proteomic data with protein-protein interaction networks (PPINs) and identified putative druggable candidates in breast cancer by analyzing topological features of the reconstructed networks (Karagoz et al., 2015; Liu et al., 2017; Li et al., 2018; Nuncia-Cantarero et al., 2018). These bioinformatics pipelines have their own power through decreasing the number of candidate therapeutic targets/drugs and proposing potential treatment strategies for subsets of breast cancer patients.

The overall prognosis for patients with basal-like breast cancer remains poor and there is an urgent need to identify molecular targets to develop effective therapeutic strategies. To take advantage of the extensive publicly available “omics” data, we integrated transcriptome with interactome data and calculated network entropy for each protein-protein interactions (PPIs) to identify the dynamic states in basal-like breast cancer. Our analyses identified modules as systems biomarkers at gene expression level and these networks were confirmed at the proteomic level. Importantly, functional annotation and analysis of module activity scores demonstrated that these modules were subtype specific. Using these models essential metabolites and drug candidates were identified within the context of basal-like specific modules. Collectively, these analyses suggest that the proposed strategy incorporating multi-omics analyses of human breast tumors has the capacity to define novel signaling networks and link these features to existing therapeutic opportunities.

**Table 1:** Validation and discovery sets used in this study.

Data type	Data portal	“Omic” level	Number of basal samples	Number of non-basal samples	Set type
Gene expression levels	TCGA	Transcriptomic	179	852	Discovery
Gene expression levels	METABRIC	Transcriptomic	331	1655	Validation
Protein expression levels	CPTAC	Proteomic	19	58	Validation
Protein expression levels	TCGA	Proteomic	160	777	Validation

## Materials and Methods

### Data Collection

Throughout the study, we integrated multi-omics data including genomics, transcriptomics, and proteomics using network analysis (Table 1). TCGA data were obtained from https://gdac.broadinstitute.org/, METABRIC and CPTAC data were collected from Supplementary Files of these studies. At transcriptomic level, gene expressions were obtained from two major initiatives presenting RNA-Seq data from the TCGA study and microarray data from the METABRIC study. Normalized gene expression values for 179 basal and 852 non-basal like breast cancer samples (*n* = 1031) from TCGA, and 331 basal and 1665 non-basal samples from the METABRIC project (*n* = 1992) were used in integrative analysis. At the protein level, two different sources were used, (i) expression data of 160 basal and 777 non-basal like samples (*n* = 937) in TCGA, using Reverse Phase Protein Array (RPPA)- based analysis of 226 proteins, and (ii) expression data of 19 basal and 58 non-basal like samples (*n* = 77) from CPTAC which performed comprehensive mass-spectrometry methods including around 10,000 proteins (Mertins et al., 2016).

RNA sequencing data from TCGA (*n* = 1031) were used as a discovery set whereas, microarray data from METABRIC and proteomic data from TCGA and/or CPTAC were used as independent validation data sets in the study (Table 1).

### Differential Interactome

To obtain a differential view of human interactome between two different phenotypes, and to identify PPIs that are up- or down-regulated in each phenotype relative to the other one, we used the gene expression profiles of interacting protein pairs and recruited the differential interactome analysis as previously described (Ayyildiz et al., 2017). For this purpose, normalized gene expression profiles from TCGA (179 basal-like and 852 non-basal like samples) were categorized into three levels: high (1), moderate (0), and low (-1) expression levels according to comparison of each gene expression with the average expression within each sample. The probability distributions for any possible co-expression profile of gene pairs (encoding proteins interacting with each other) were estimated, and the uncertainty of determining whether or not a PPI in encountered in a phenotype was estimated through an entropy formulation. In order to define possible PPIs, we used the high confidence human PPIs (Karagoz et al., 2016), comprising 147,923 interactions among 13,213 proteins. Karagoz and coworkers assembled and integrated physical PPIs of Homo sapiens from six publicly available databases including BioGRID (Chatr-Aryamontri et al., 2015), DIP (Salwinski, 2004), IntAct (Orchard et al., 2014), HIPPIE (Schaefer et al., 2012), HomoMINT (Persico et al., 2005), and HPRD (Prasad et al., 2009). Then, PPIs analyzed the differential view of human interactome between the basal and non-basal subtypes of breast cancer; *P* < 0.05 was considered statistically significant for these analyses.

### Differentially Expressed Genes and Proteins

Both differentially expressed genes (DEGs) between 179 basal and 852 non-basal samples in TCGA cohort, and differentially expressed proteins (DEPs) between 19 basal and 61 non-basal samples in CPTAC cohort were identified by using the Significance Analysis of Microarrays (SAM) method implemented in R software (Tusher et al., 2001; Hu et al., 2016; Gámez-Pozo et al., 2017). False Discovery Rate (FDR), adjusted *p*-value was set at *p* < 0.05, and fold changes > 1 between basal-like and non-basal samples were considered as up-regulated DEGs and proteins in basal tumors.

### Module Extraction From Basal Specific Networks

Basal subtype specific PPI networks were constructed by using the differential interactome from basal-like tumors. The interactions associated with proteins corresponding to DEGs that are up-regulated in basal-like tumors were identified and used to construct up-regulated PPI networks specific to basal-like breast cancer. The networks were visualized by using Cytoscape software (version 3.4.0) (Lopes et al., 2011). The topological analysis of the networks was performed via CytoNCA plugin of Cytoscape (version 2.1) (Tang et al., 2015). Two different topological metrics, degree, which is defined by the number of adjacent nodes of a node in the network, and betweenness centrality, which characterizes nodes by how often they occur on the shortest path between two other nodes in the network, were simultaneously employed to define hub nodes. Hub nodes with higher degree or betweenness values were reported to have significant roles in cellular signal trafficking and could be potential candidate biomarkers or drug targets Modules were identified as highly connected subnetworks within up-regulated networks. Gene expression data from METABRIC were used for validation of the gene expression modules in basal-like breast cancer.

### Functional Annotation

Functional enrichment analysis associated with the three protein-protein interaction modules were analyzed using QIAGEN’s Ingenuity^®^Pathway Analysis (IPA^®^, QIAGEN Redwood City) ^1^.

### Module Activity

In order to convert the identified EED, AURKA, and DHX9 modules to gene expression signatures that can be used to quantify pathway activity in a given sample from independent datasets, the module was converted to a gene list and the mean expression of unweighted gene list was used to calculate a pathway score. For these studies, a score was calculated for each sample in the TCGA (discovery) and METABRIC cohort (validation). Analysis of variance (ANOVA) tests were used to quantify differences between the EED-module, DHX9-module and AURKA-module activity scores between breast cancer subtypes in each dataset. A Student’s *t*-test was used to evaluate levels of EED, DHX9e and AURKA signature scores between adjacent normal breast tissue and basal-like tumors. To infer the functional roles of these modules, a panel of 270 experimentally derived gene expression signatures that predict activation of various oncogenic signaling pathways, was performed by integrating gene expression data as described previously (Gatza et al., 2014). To identify the association of the modules with oncogenic pathways, a Spearman’s rank correlation was used between oncogenic pathway activity scores and EED, DHX9 and AURKA activity scores.

### Module Specific Drug Repositioning

To identify small molecules that can potentially reverse gene expression of basal-like tumors, we utilized the Library of Integrated Network-based Cellular Signatures (LINCS) – L1000 data which includes gene expression data from ∼50 human cell line in response to ∼20,000 compounds (Campillos et al., 2008). We queried basal-like specific module genes which are all up-regulated and down-regulated DEGs (Fold Change < 0.2) signatures as input. We used the L1000CDS2 (Duan et al., 2016) search engine, which contains 30,000 significant signatures that were processed from the LINCS L1000 data, to identify small molecule signatures associated with each module. The identified drugs were ranked based on their scores and the top 50 were acquired for each query. Drugs were checked through literature review and publicly available datasets such as CTD (Davis et al., 2017) and KEGG DRUG (Kanehisa et al., 2012) to identify those that were previously investigated within the context of breast cancer.

### Subtype Specific Essential Metabolites

We next acquired 917 personalized genome scale metabolic models (GEMs) of breast cancer patients (Uhlen et al., 2017). We analyzed each patient GEM to identify essential metabolites for tumor growth by removing the reactions in which the metabolite functions as substrate regardless of compartmentalization (Bidkhori et al., 2018). Next, we categorized personalized models based on clinical information to create subtype-specific patient metabolic models and found the percentage of subtype representation of each metabolite. A Fisher exact test was applied to identify statistically significant difference between basal-like and non-basal-like (i.e., all other tumors) for each metabolite. Significant difference between subtypes was determined based on a *P* < 0.05.

## Results

### Basal-Like Subtype Specific PPI Elucidation via Differential Interactome

Cancer cells are characterized by increase in network entropy comprising high uncertainty, pathway redundancy and promiscuous signaling resulting from intra-sample heterogeneity. Recently, a differential interactome network analysis were presented to show the uncertainties of PPIs in ovarian cancer (Ayyildiz et al., 2017). In this study, we employed differential interactome algorithm utilizing the entropy concept using a comprehensive gene expression data and human PPI network to reveal the heterogeneity among the breast cancer subtypes (i.e., basal-like vs. non-basal-like). To do so, we categorized the expression of each gene and for each patient using 179 basal and 852 non-basal-like samples from TCGA into three classes as -1, 0, 1, These classes were then integrated with a high confident PPI network (Karagoz et al., 2016) and the frequency of PPIs estimated for both basal-like and non-basal-like tumors. Using a 95% confidence interval (*p* < 0.05), significant values <0.2 and >0.8 as well as corresponding *H* < 0.7 were calculated for each class. As a result, 3,002 interactions among 1,652 proteins were considered significant across the entire dataset. These analyses identified 2,291 interactions among 1,391 proteins as being significantly activated in basal-like tumors whereas 712 interactions among 612 proteins were identified as significant in non-basal-like samples; 351 proteins were common across both subgroups of tumors (Supplementary Table S1).

**FIGURE 1:**
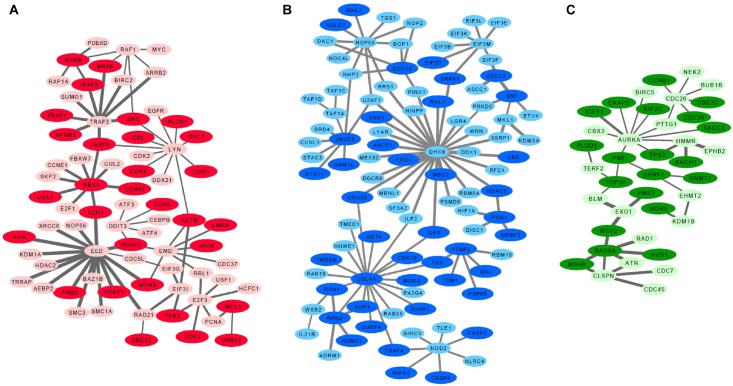
Basal like breast cancer specific highly connected protein-protein interaction modules. **(A)** EED module, **(B)** DHX9 module, **(C)** AURKA module. Darker nodes indicate the statistically significant positive correlations between mRNA and protein pairs. Thicker edges indicate lowest entropy levels between interacting pairs.

Since low entropy presents low uncertainty, low redundancy and deterministic signaling resulting with homogeneity in the population, we next focused on the basal-like subtype to identify low entropy interactions (*H* < 0.1). These analyses identified the EED protein network which is defined by 82 interactions within the group of 98 proteins. Importantly, the lowest entropy profile of the EED centroid network only identified an interaction with one protein (CTCF) in non-basal-like tumors. We further identified a sub-set of proteins, excluding 351 common signatures evident in both basal-like and non-basal-like tumors to identify a basal-like subtype specific network (Supplementary Table S2). All differential interactome networks and basal-like subtype specific networks were delimited regarding up-regulated genes in the basal-like tumors through 2-class SAM analysis (Tusher et al., 2001; Supplementary Table S3). Through the integration of SAM analysis and the above detailed differential interactome framework, we identified three significant modules: EED centroid module, covering relatively low entropy PPIs (Figure 1A); the DHX9 centroid module, covering mixed of low and high entropy PPIs (Figure 1B); and the AURKA centroid module, covering relatively high entropy PPIs (Figure 1C).

Further analyses of the EED, DHX9, and AURKA modules determined that genes included in EED-module play roles in cyclins and cell cycle regulation (*p* = 6.1e-19), cell cycle: G1/S checkpoint regulation (*p* = 3.5e-18), regulation of cellular mechanics by calpain protease (*p* = 1.6e-11), aryl hydrocarbon receptor signaling (*p* = 4.3e-11), apoptosis signaling (*p* = 7.0e-10), TWEAK signaling (*p* = 1.8e-09), and GADD45 signaling (*p* = 4.3e-9), In contrast, the genes in DHX9-module contribute to mTOR signaling (*p* = 4.1e-06), regulation of eIF4 and p70S6K signaling (*p* = 7.9e-06), EIF2 signaling (*p* = 7.2e-05), Inflammasome pathway (*p* = 1.4e-04), assembly of RNA Polymerase I Complex (*p* = 1.1e-03), DNA double strand break repair (*p* = 1.8e-03) and cell cycle (*p* = 3.5e-03) while the genes associated with the AURKA-module are involved in DNA damaged-induced 14-3-3A signaling (*p* = 1.8e-10), mitotic roles of Polo like kinase (*p* = 2.1e-09), role of CHK proteins in cell cycle checkpoint control (*p* = 6.0e-08), ATM signaling (*p* = 9.3e-07) and mismatch repair (*p* = 3.1e-06), role of BRCA1 in DNA damage response (*p* = 1.3e-05), and cell cycle (*p* = 9.8e-05). These data suggest that each module represent a unique aspect of basal-like breast cancer signaling. Some of these pathways such as TWEAK signaling, apoptosis signaling, mTOR signaling, ATM signaling showed that the chemotherapy targeted pathways are also activated in basal-like tumors in which chemotherapy is the front-line treatment option, nowadays (Supplementary Figure S1).

### Proteomic Analysis of Basal Specific Modules

We next reconstructed PPI networks using transcriptome data and validated our findings at proteomic level by leveraging orthogonal genomic and proteomic data from the TCGA and CPTAC projects. Transcriptome data from 937 sample was compared to RPPA analysis of the same samples to assess the relationship between each network at the 226 proteins and phosphoproteins from TCGA. Likewise the gene expression data from a subset of 77 of these samples was used to examine the relationship between each module and 10,062 proteins and phosphoproteins using mass spectrometry-derived proteomic data from the CPTAC project. First, we used CPTAC proteome data to compare each gene to its corresponding protein across all basal-like tumors and assessed correlation for those pairs. Overall, 52.6–64.5% of the mRNA-protein pairs showed statistically significant positive Spearman correlations (*P* < 0.05) when changes in mRNA abundance were compared to changes in relative protein abundance. These proteins in basal-like samples are shown in darker colors in Figure 1A–C. Then, we identified DEPs between basal-like and non-basal-like samples by using both RPPA and CPTAC data. Although RPPA data has limited number of proteins, we identified several up-regulated proteins including CCNE1, RAF1, SRC, CDK1, EGFR, MYC, MYH9, PCNA associated with the EED-module. Similarly, NDGR1 and CCNB1 were associated with the DHX9 and AURKA modules, respectively. We also analyzed DEPs between basal-like and non-basal-like tumors by using CPTAC data which is more comprehensive than RPPA data and it covered 69.4–56.4% of the module genes and 29.4–36.4% of these proteins were identified as being up-regulated in basal-like tumors (Supplementary Table S3).

### Modules as Basal Specific Signatures

**FIGURE 2:**
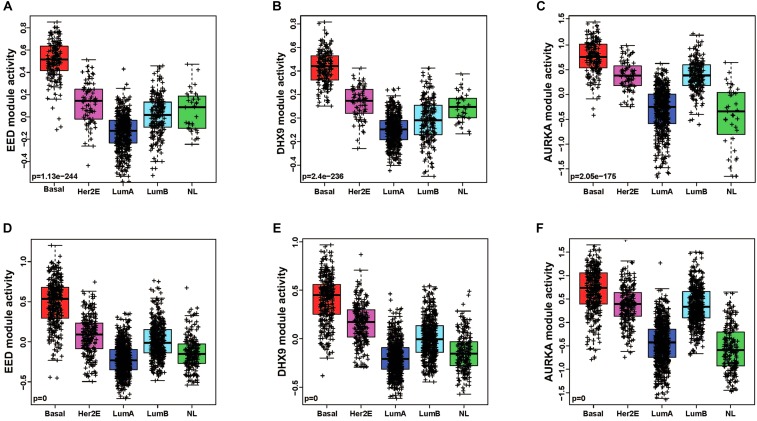
The pattern of basal like breast cancer specific modules activity across breast cancer subtypes. **(A–C)** EED, DHX9, and AURKA modules are highly activated in basal like tumors by using TCGA cohort-discovery set. **(D–F)** EED, DHX9, and AURKA modules are highly activated in basal like tumors by using METABRIC cohort-validation set.

**FIGURE 3:**
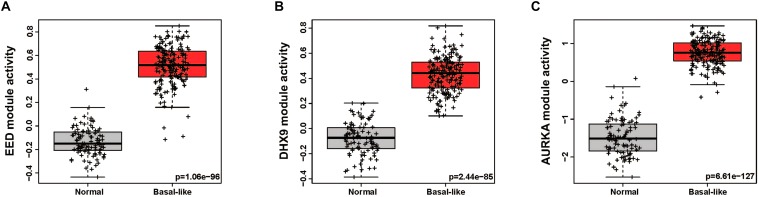
The activity levels of basal like breast cancer specific modules in normal and basal like tumors. **(A)** EED module, **(B)** DHX9 module, **(C)** AURKA module.

In order to quantitatively assess the activity of each modular in each patient sample, we next generated a gene expression signature on the basis of median expression of each gene in the module. This strategy was used to calculate a module score for each sample in the TCGA (discovery set) and METABRIC (validation set) datasets. We then quantitatively evaluated the differences in the module activities across breast cancer subtypes by an ANOVA test. These analyses demonstrated that EED (*P* = 1.13e-244), DHX9 (*P* = 2.4e-236), and AURKA (*P* = 2.05e-175) activity was highest in basal-like tumors in the TCGA cohort (Figure 2A–C); these findings were validated by analysis of module activity in the METABRIC cohort (Figure 2D–F). Finally, we determined that the EED (*P* = 1.06-e96), DHX9 (*P* = 2.44e-85), and AURKA modules (*P* = 6.61e-127) were expressed at significantly higher levels in in basal-like tumors compared to adjacent normal tissue (Figure 3A–C).

### Functionality of Basal Specific Modules

**FIGURE 4:**
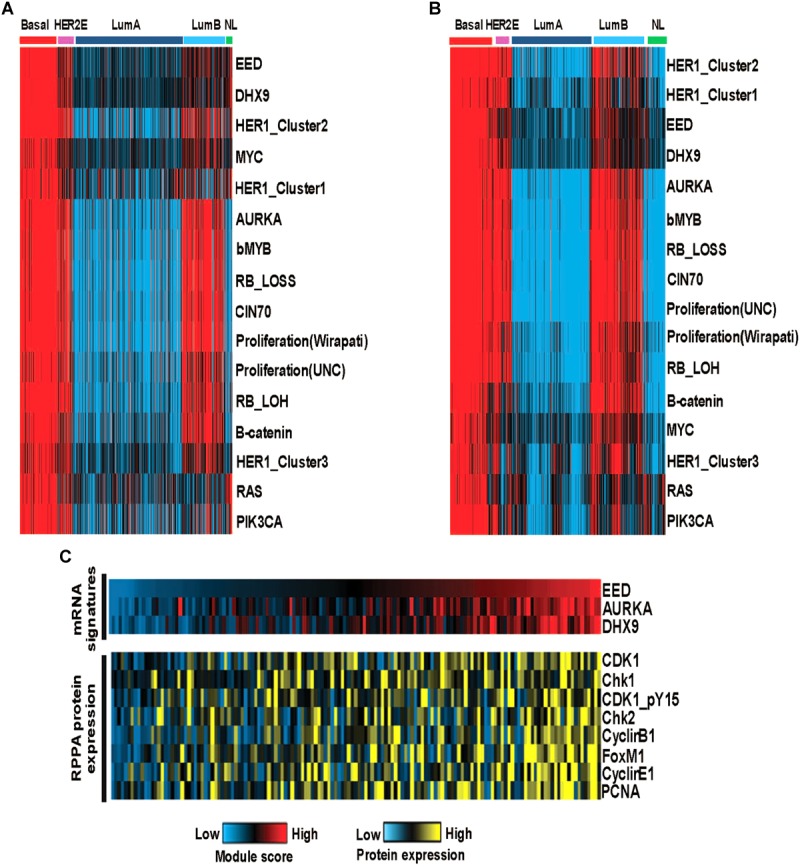
The functional analysis of basal like breast cancer specific modules **(A)** The activity of oncogenic pathways correlated with module activities in TCGA cohort-discovery set. **(B)** The activity of oncogenic pathways correlated with module activities in METABRIC cohort-validation set. **(C)** High module activities characterized by high expression of cell cycle proteins.

We examined the functional roles of these modules by exploring the correlations with a series of previously published gene expression signatures which are capable of measuring oncogene or tumor suppressor pathway activity, aspects of the tumor microenvironment and other tumor characteristics. We identified pathway activities, which were positively (or negatively) correlated with module activities using a Spearman rank correlation to assess the relationship between pathway activity and the EED, DHX9, or AURKA module activity scores. As expected, our data recapitulated known characteristics of basal-like tumors including low hormone receptor signaling and high expression of proliferation pathway activity and demonstrated the relationship between these characteristics and the expression of each module (i.e., EED, DHX9, and AURKA). Moreover, these modules were associated with multiple indicators of proliferation including, RB_LOSS, RB_LOH, and bMYB highly correlated with these module activities as well as RAS, PIK3CA, β-catenin, MYC and HER1_Cluster 1, HER1_Cluster 2, and HER1_Cluster 3 signatures (Figure 4A). Consistent results were obtained using the METABRIC data (Figure 4B). Importantly, we also confirmed the ability of the transcriptomic module signatures to assess the functional roles of EED, DHX9, and AURKA modules by exploring relationships between the module signature scores and protein expression. Analysis of RPPA data from basal-like samples confirmed that these tumors with high module scores have significantly higher levels of CHK1, CHK2, CDK1, Cyclin B1, Cyclin E1, FOXM1, and PCNA protein expression consistent with their role in cell cycle regulation and proliferation (Figure 4C).

### Drug Repositioning Based on Basal Subtype Specific Modules

As discussed above, the EED, DHX9, and AURKA modules were converted to gene expression signatures on the basis of up-regulated genes specific to each module; as would be expected down-regulated genes (Fold Change < 0.2) were common for all modules. We asked the question of whether each module/signature identified potential therapeutic opportunities. To do so, we queried each gene signatures separately against the LINCS database L1000CDS2 (Duan et al., 2016) in order to identify concordant and discordant patterns of gene expression between each module and gene expression profiles associates with drug-induced and/or disease expression. Drugs that resulted in a gene expression profile that was negatively correlated with each module were identified and selected as potential candidate compounds that had the potential to reverse the activity of each module network that was associated with basal-like tumors (Supplementary Figure S2). Since we have demonstrated specificity of the modules to basal-like tumors, we may also propose that our candidate drugs are specifically targeting basal-like tumors.

**FIGURE 5:**
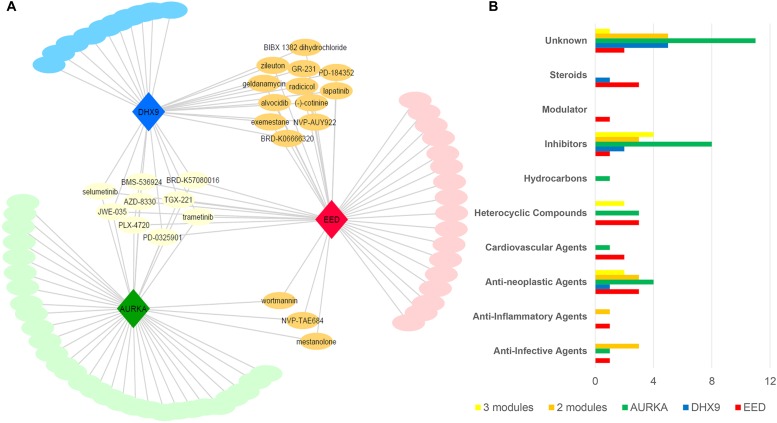
Drug repositioning for basal like breast cancer specific modules **(A)** Module-drug networks **(B)** Drug categories of module specific drugs and common drugs among modules.

After removing the duplicated drugs from query results, we found that EED and AURKA modules were associated with 41 candidate compounds while DHX9 was associated with 31 candidate small molecules. Networks comprising drug candidates and modules were found to have 114 interaction between three modules and 80 drugs (Figure 5A). The 80 identified drugs were categorized as molecular inhibitors (23%), anti-neoplastic agents (15%), heterocyclic compounds (10%), anti-infective agents (6%), or steroids (6%). Moreover, a number of the drugs specific to each module (as well as some common candidates) were also identified in each drug category (Figure 5B). There are at least 19 approved, 24 investigational, and 6 experimental drugs listed in DrugBank (version 5.1.1), however there are perturbagens used in L1000 platform without detailed information (Supplementary Table S4).

**Table 2:** Various drug candidates that already associated with breast cancer via different sources.

Drug name	Literature evidence	CTD	KEGG drug	Clinical trials
Epirubicin	Warm et al., 2010	✓	✓	NCT00176488
Erlotinib	Catania et al., 2006			NCT01650506
Lapatinib	Giampaglia et al., 2010	✓		NCT00694252
Exemestane	Goss et al., 2013	✓	✓	NCT00810797
Wortmannin	Li et al., 2012	✓		
Alvocidib	Murphy and Dickler, 2015	✓		NCT00039455
Tyrphostin ag 1478	Zhang et al., 2008	✓		
Canertinib	Gschwantler-Kaulich et al., 2016	✓		NCT00051051
Danazol	Coombes et al., 1983	✓		
Palbociclib	Finn et al., 2016	✓	✓	NCT02513394

Nine of the drugs including selumetinib, trametinib, and several other investigational drugs were common to each of the three modules. Consistent with our results, selumetinib as MEK inhibitor was reported to suppresses cell proliferation, migration, and trigger apoptosis, following G1 arrest in TNBC cells (Zhou et al., 2016). Furthermore, the MEK inhibitor, trametinib is also a therapy of significant interest for the treatment of TNBC since TNBC cell lines have been shown to be especially sensitive to this drug (Jing et al., 2012; Davis et al., 2014). Finally, we noted some overlap between drugs associated with each module. For instance, the three common drugs (i.e., wortmannin, mestanolone, NVP-TAE684) are associated with both the EED and AURKA modules while 12 drugs (i.e., radicicol, lapatinib, alvocidib, zileuton, geldanamycin, exemestane) are consistent between the EED and DHX9 module (Supplementary Table S4). Intriguingly, 10 of our candidate drugs were previously associated with the breast cancer based on at least one of the sources including CTD, KEGG Drug, Clinical Trials, and scientific literature (Table 2).

Since EED module has the lowest entropy level between PPIs, we focused on 17 drug candidates which are only related to EED module in addition to common drugs. Three of these drugs are anti-neoplastic agents and five of them are unknown, however, others belonged to steroids (BRD-A94793051, Oxymetholone, Testosterone propionate), PLK inhibitor (BI-2536), heterocyclic compounds (BRD-K17953061, GDC-0980, TG101348), cardiovascular agents (BRD-K52080565, S-2500), anti-inflammatory (oxaprozin), and anti-infective agents (5-fluorocytosine).

### Essential Metabolites and Anti-metabolites as Drug Candidates

**FIGURE 6:**
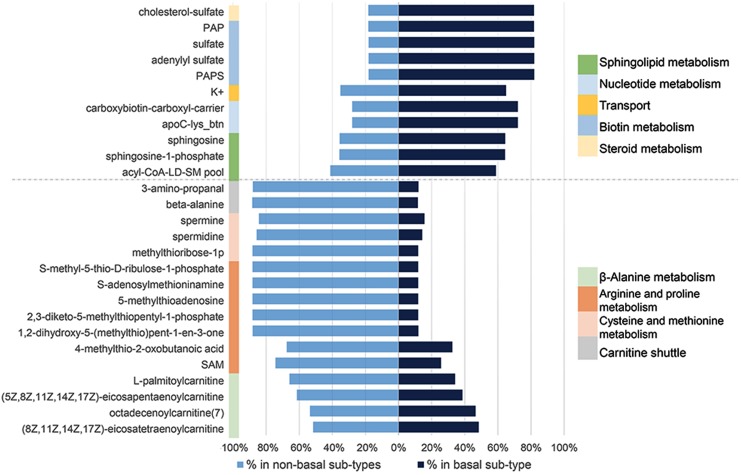
Significant essential metabolite differences between non-basal and basal like breast cancer specific personalized metabolic models and their associated pathways.

GEMs reconstructed for different cancer tissues have been used for characterization of metabolic modifications; disease stratification and determination of drug targets using essential genes or metabolites (Folger et al., 2011; Agren et al., 2012; Bidkhori et al., 2018). To address this question, we first identified a panel of 917 personalized GEMs derived from breast cancer patients (Uhlen et al., 2017). We then categorized each GEMs based on clinical information to create subtype-specific patient metabolic models. These models were then used to identify subtype-specific metabolites essential for tumor growth. After categorization of BCS, percentage of abundance for each essential metabolite was calculated. Significant alteration between the abundance of basal-like and non-basal BCS were determined based on FDR adjusted P-value threshold (P-adj < 0.05) (Supplementary Table S5). These analyses identified 27 essential metabolites (Supplementary Table S6); 11 were significantly enriched in basal-like tumors while the remaining 16 were enriched in non-basal-like samples. Further analyses determined that the essential metabolites that are expressed at higher levels in basal-like tumors were associated with steroid metabolism, biotin metabolism, nucleotide metabolism, sphingolipid metabolism and transport. Conversely, the identified metabolites down-regulated in basal-like samples were involved in beta-alanine metabolism, arginine and proline metabolism, cysteine and methionine metabolism, and carnitine shuttle (Figure 6).

## Discussion

The dynamics of cells are regulated by PPIs and properties of networks such as entropy provide information about the current state of the network. Given that cancer cells are reported to have an increase in network entropy, several previous studies have integrated gene expression data with PPI network information to compute the energetic state of cancer cells by calculating entropy (West et al., 2012; Teschendorff et al., 2015; Rietman et al., 2016). Likewise, a number of studies have used a network-based entropy approach to identify disease specific PPIs as biomarker candidates, proliferative and prognostic markers in lung and breast cancer, as well as to demonstrate the association between network entropy and tumor initiation, progression, and anticancer drug responses (Varadan and Anastassiou, 2006; Xiong et al., 2010; Banerji et al., 2013; Lecca and Re, 2015; Cheng et al., 2016; Ayyildiz et al., 2017).

The current study employed a novel multi-omics-based approach to integrate genomic, proteomic and metabolomic tumor data. Our analyses of mRNA expression data identified three highly connected modules which are centered on the activation of the EED, DHX9, and AURKA signaling networks. These data demonstrated that each module is highly activated in basal-like tumors compared to non-basal-like tumors as well as adjacent normal tissues. Importantly, by analyzing proteome data, our results confirmed the correlation between the expression of genes and proteins that comprise each identified module. By analyzing the association between module expression and oncogenic signaling using a panel of more than 250 gene expression signatures, we were able to assess the functional relationship of these modules with known oncogenic and signaling features. Our results demonstrated the correlation between EED, DHX9, and AURKA module activity and proliferative oncogenic pathways including RAS, PI3K, and Rb/E2F signaling in basal-like tumors. Consistent with these results, CHK1, CHK2, CDK1, Cyclin B1, Cyclin E1, and PCNA protein expression levels were identified higher in tumors with high module scores. Through integrated analyses, we identified candidate drugs to target three modules by drug repositioning. Utilizing multiple omics data including genome, transcriptome, and interactome, we repurposed 519 agents for breast cancer by incorporating data from the LINCS project (Duan et al., 2016) into our analyses. In another drug repositioning study, five of the identified repurposed candidate agents showed superior therapeutic indices compared to doxorubicin in *in vitro* assays in basal sub-type cell line (SUM149) in addition to luminal cell line (MCF7) (Chen et al., 2016). Moreover, Lee et al. (2016) developed an integrative approach for drug repositioning using the expression signature, chemical structure, target signatures and LINCS data. They applied this strategy to identify candidate anti-cancer drugs for breast cancer (Lee et al., 2016). Although there are previous computational drug-repositioning efforts that utilized LINCS as mentioned, the methodologies are focused on breast cancer regardless of disease heterogeneity and subtype information.

In addition, our analyses identified subtype-specific metabolites, including several specific to basal-like tumors, which may provide opportunity to design anti-metabolite drugs for breast cancer. Results in essential metabolite analysis emphasized sphingolipids and steroid metabolism for basal-like breast cancer. Sphingolipid levels in breast cancer tissue are generally higher than normal breast tissue and bioactive sphingolipids, such as sphingosine-1-phosphate (S1P) has many cellular functions like cell proliferation, migration, survival, immune cell trafficking, and angiogenesis which are related to cancer progression and metastasis (Nagahashi et al., 2016). However, sphingosine and S1P were recently highlighted as important for signaling mechanisms in metastatic TNBC and its targeted therapy (Maiti et al., 2017). A recent lipidomics profiling of TNBC tumors also supported sphingolipids as potential prognostic markers and associated enzymes as candidate therapeutic targets (Purwaha et al., 2018) in parallel to our results.

TNBC was associated with expression pattern of 2-pore domain potassium (K2p) channels which enable background leak of potassium (K^+^). Differential expression on K2p-channels may be suggested as a novel molecular marker related to potassium levels in basal like BCS (Dookeran et al., 2017). In another study, expression of calcium-activated potassium (SK4) channels were also associated with TNBC and cellular functions such as proliferation, migration, apoptosis, and EMT processes (Zhang et al., 2016).

Breast cancer is known as one of the malignancies in which steroid hormones drive cellular proliferation (Capper et al., 2017). As steroid metabolism associated metabolite, cholesterol sulfate, is quantitatively the most important known sterol sulfate in human plasma and may play a role in cell adhesion, differentiation and signal transduction (Strott and Higashi, 2003). Given that current standard-of-care therapy for TNBC is largely limited to multi-agent cytotoxic chemotherapy, the potential of incorporating identified repurposed drugs and/or targeting identified modules and/or metabolites represents a potential therapeutic opportunity for a subset of patents with limited treatment options.

Given these data, we would propose that the strategy outlined here can be used to repurposed drugs in order to identify novel candidate compounds or drugs to be utilized in not only monotherapy but also in combination therapy for the treatment of TNBC. Consistent with this argument, a number of the candidate drugs identified by our analyses have been incorporated in ongoing clinical trials. For instance, TNBC patients who received pre-operative sequential epirubicin and cyclophosphamide followed by docetaxel were found to have a significant increase in pathological complete response (PCR) (Warm et al., 2010). Although a great number of pre-clinical trials will be necessary to support the *in silico* modeling detailed in the current study prior to initiation of clinical trials, a large number of identified candidates have significant *in vitro* and *in vivo* support to indicate that these represent potential therapeutic opportunities. For instance, drugs inhibiting cyclin-dependent kinases (CDKs), including the CDK9 inhibitor alvocidib have been reported to be effective against TNBC (Ocana and Pandiella, 2015).

Erlotinib also showed anti-tumor effect on TNBC in a xenograft model (Ueno and Zhang, 2011). Likewise, targeting the MET and EGFR receptors, which regulate RAS/ERK and PI3K/AKT signaling, resulted in improved treatment compared to monotherapy (Linklater et al., 2016).

The current study has defined a novel approach to identify breast cancer subtype-specific network modules via a network entropy-based approach. This strategy can be used for both the identification of potentially novel signaling networks but also to identify subtype-specific therapeutic opportunities through drug repositioning. Importantly, we demonstrate that this approach can be used to link signaling networks with and subtype-specific essential metabolites which represents additional therapeutic opportunities. As such, the current studies have the potential enhancing the impact of existing therapeutics or multi-agent therapeutic strategies by identifying novel drug/target networks in the context of breast cancer and in breast cancer subtypes. On a broader scale, this strategy is largely applicable to all cancer and disease type/subtypes where multi-platform genomic, proteomic, and metabolomic data exists and thus represents a potential strategy to define novel signaling networks unique to each disease and identify disease/subtype-specific therapeutic strategies.

## Author Contributions

BT and KK designed the study, performed the all other analyses, and wrote the manuscript. GB performed essential metabolite analysis. MG, RS, AM, MU, and KA supervised the work and contributed to the manuscript during the progress of the work. All authors reviewed and approved the final manuscript.

## Conflict of Interest Statement

The authors declare that the research was conducted in the absence of any commercial or financial relationships that could be construed as a potential conflict of interest.

## Footnotes

1 www.qiagen.com/ingenuity

## Supplementary Material

FIGURE S1 Functional enrichment results of the genes involved in each basal-like module using Ingenuity Pathway Analysis (IPA).

FIGURE S2 The gene signatures of three modules separately on L1000CDS2 for elucidating the differences and similarities between drug-induced expression profiles and disease expression. Drugs were ranked for each module and we elected drugs that showed negatively correlated action mechanisms with the module gene signatures to reverse disease gene expression.

TABLE S1 Non-basal and basal-like subtype specific PPI elucidation via differential interactome.

TABLE S2 Three modules for only in basal-like subtype specific networks.

TABLE S3 Statistical values of differential expressed genes and proteins.

TABLE S4 Information of repurposed module specific and common drug signatures.

TABLE S5 Essential metabolite and personalized model matrix and breast cancer categorization of personalized GEMs.

TABLE S6 Significant essential metabolites between non-basal and basal-like breast cancer.

## References

[B1] Agren R. Bordel S. Mardinoglu A. Pornputtapong N. Nookaew I. Nielsen J. (2012). Reconstruction of genome-scale active metabolic networks for 69 human cell types and 16 cancer types using INIT. *PLoS Comput. Biol.* 8:e1002518. 10.1371/journal.pcbi.1002518 22615553 PMC3355067

[B2] Ayyildiz D. Gov E. Sinha R. Arga K. Y. (2017). Ovarian cancer differential interactome and network entropy analysis reveal new candidate biomarkers. *Omi. A J. Integr. Biol.* 21 285–294. 10.1089/omi.2017.0010 28375712

[B3] Banerji C. R. S. Miranda-Saavedra D. Severini S. Widschwendter M. Enver T. Zhou J. X. (2013). Cellular network entropy as the energy potential in Waddington’s differentiation landscape. *Sci. Rep.* 3:3039. 10.1038/srep03039 24154593 PMC3807110

[B4] Bidkhori G. Benfeitas R. Elmas E. Kararoudi M. N. Arif M. Uhlen M. (2018). Metabolic network-based identification and prioritization of anticancer targets based on expression data in hepatocellular carcinoma. *Front. Physiol.* 9:916. 10.3389/fphys.2018.00916 30065658 PMC6056771

[B5] Campillos M. Kuhn M. Gavin A. C. Jensen L. J. Bork P. (2008). Drug target identification using side-effect similarity. *Science* 321 263–266. 10.1126/science.1158140 18621671

[B6] Capper C. P. Rae J. M. Auchus R. J. (2017). The metabolism, analysis, and targeting of steroid hormones in breast and prostate cancer. *Horm Cancer* 7 149–164. 10.1007/s12672-016-0259-0 26969590 PMC4860032

[B7] Catania C. De Pas T. M. Pelosi G. Manzotti M. Adamoli L. Nolè F. (2006). Erlotinib-induced breast cancer regression. *Ann. Pharmacother.* 40 2043–2047. 10.1345/aph.1H252 17062833

[B8] Chatr-Aryamontri A. Breitkreutz B. J. Oughtred R. Boucher L. Heinicke S. Chen D. (2015). The BioGRID interaction database: 2015 update. *Nucleic Acids Res.* 43 D470–D478. 10.1093/nar/gku1204 25428363 PMC4383984

[B9] Chen H.-R. Sherr D. H. Hu Z. DeLisi C. Jin G. Fu C. (2016). A network based approach to drug repositioning identifies plausible candidates for breast cancer and prostate cancer. *J. Natl. Compr. Canc. Netw.* 8 1–21. 10.1186/s12920-016-0212-7 27475327 PMC4967295

[B10] Cheng F. Liu C. Shen B. Zhao Z. (2016). Investigating cellular network heterogeneity and modularity in cancer: a network entropy and unbalanced motif approach. *BMC Syst. Biol.* 10:65. 10.1186/s12918-016-0309-9 27585651 PMC5009528

[B11] Ciriello G. Gatza M. L. Beck A. H. Wilkerson M. D. Rhie S. K. Pastore A. (2015). Comprehensive molecular portraits of invasive lobular breast cancer. *Cell* 163 506–519. 10.1016/j.cell.2015.09.033 26451490 PMC4603750

[B12] Coombes R. C. Perez D. Gazet J.-C. Ford H. T. Powles T. J. (1983). Danazol treatment for advanced breast cancer. *Cancer Chemother. Pharmacol.* 10 194–195. 10.1007/BF00255761 6345017

[B13] Curtis C. Shah S. P. Chin S. F. Turashvili G. Rueda O. M. Dunning M. J. (2012). The genomic and transcriptomic architecture of 2,000 breast tumours reveals novel subgroups. *Nature* 486 346–352. 10.1038/nature10983 22522925 PMC3440846

[B14] Davis A. P. Grondin C. J. Johnson R. J. Sciaky D. King B. L. McMorran R. (2017). The comparative toxicogenomics database: update 2017. *Nucleic Acids Res.* 45 D972–D978. 10.1093/nar/gkw838 27651457 PMC5210612

[B15] Davis S. L. Eckhardt S. G. Tentler J. J. Diamond J. R. (2014). Triple-negative breast cancer: bridging the gap from cancer genomics to predictive biomarkers. *Ther. Adv. Med. Oncol.* 6 88–100. 10.1177/1758834013519843 24790649 PMC3987651

[B16] Dookeran K. A. Zhang W. Stayner L. Argos M. (2017). Associations of two-pore domain potassium channels and triple negative breast cancer subtype in the cancer genome atlas: systematic evaluation of gene expression and methylation. *BMC Res. Notes* 10:475. 10.1186/s13104-017-2777-2774 28899398 PMC5596847

[B17] Duan Q. Reid S. P. Clark N. R. Wang Z. Fernandez N. F. Rouillard A. D. (2016). L1000CDS2: LINCS L1000 characteristic direction signatures search engine. *NPJ Syst. Biol. Appl.* 2:16015. 10.1038/npjsba.2016.15 28413689 PMC5389891

[B18] Finn R. S. Martin M. Rugo H. S. Jones S. Im S.-A. Gelmon K. (2016). Palbociclib and letrozole in advanced breast cancer. *N. Engl. J. Med.* 375 1925–1936. 10.1056/NEJMoa1607303 27959613

[B19] Folger O. Jerby L. Frezza C. Gottlieb E. Ruppin E. Shlomi T. (2011). Predicting selective drug targets in cancer through metabolic networks. *Mol. Syst. Biol.* 7 1–10. 10.1038/msb.2011.35 21694718 PMC3159974

[B20] Gámez-Pozo A. Trilla-Fuertes L. Berges-Soria J. Selevsek N. López-Vacas R. Díaz-Almirón M. (2017). Functional proteomics outlines the complexity of breast cancer molecular subtypes. *Sci. Rep.* 7:10100. 10.1038/s41598-017-10493-w 28855612 PMC5577137

[B21] Gatza M. L. Silva G. O. Parker J. S. Fan C. Perou C. M. (2014). An integrated genomics approach identifies drivers of proliferation in luminal-subtype human breast cancer. *Nat. Genet.* 46 1051–1059. 10.1038/ng.3073 25151356 PMC4300117

[B22] Giampaglia M. Chiuri V. E. Tinelli A. De Laurentiis M. Silvestris N. Lorusso V. (2010). Lapatinib in breast cancer: clinical experiences and future perspectives. *Cancer Treat. Rev.* 36(Suppl. 3), S72–S79. 10.1016/S0305-7372(10)70024-4 21129615

[B23] Goss P. E. Ingle J. N. Pritchard K. I. Ellis M. J. Sledge G. W. Budd G. T. (2013). Exemestane versus anastrozole in postmenopausal women with early breast cancer: NCIC CTG MA.*27* - A randomized controlled phase III trial. *J. Clin. Oncol.* 31 1398–1404. 10.1200/JCO.2012.44.7805 23358971 PMC3612593

[B24] Gschwantler-Kaulich D. Grunt T. W. Muhr D. Wagner R. Kölbl H. Singer C. F. (2016). HER specific TKIs exert their antineoplastic effects on breast cancer cell lines through the involvement of STAT5 and JNK. *PLoS One* 11:e0146311. 10.1371/journal.pone.0146311 26735495 PMC4703392

[B25] Hu J. Ye F. Cui M. Lee P. Wei C. Hao Y. (2016). Protein profiling of bladder urothelial cell carcinoma. *PLoS One* 11:e0161922. 10.1371/journal.pone.0161922 27626805 PMC5023150

[B26] Jing J. Greshock J. Holbrook J. D. Gilmartin A. Zhang X. McNeil E. (2012). Comprehensive predictive biomarker analysis for MEK inhibitor GSK1120212. *Mol. Cancer Ther.* 11 720–729. 10.1158/1535-7163.MCT-11-0505 22169769

[B27] Kanehisa M. Goto S. Sato Y. Furumichi M. Tanabe M. (2012). KEGG for integration and interpretation of large-scale molecular data sets. *Nucleic Acids Res.* 40 109–114. 10.1093/nar/gkr988 22080510 PMC3245020

[B28] Karagoz K. Sevimoglu T. Arga K. Y. (2016). Integration of multiple biological features yields high confidence human protein interactome. *J. Theor. Biol.* 403 85–96. 10.1016/j.jtbi.2016.05.020 27196966

[B29] Karagoz K. Sinha R. Arga K. Y. (2015). Triple negative breast cancer: a multi-omics network discovery strategy for candidate targets and driving pathways. *OMICS* 19 115–130. 10.1089/omi.2014.0135 25611337

[B30] Kibble M. Saarinen N. Tang J. Wennerberg K. Mäkelä S. Aittokallio T. (2015). Network pharmacology applications to map the unexplored target space and therapeutic potential of natural products. *Nat. Prod. Rep.* 32 1249–1266. 10.1039/c5np00005j 26030402

[B31] Lecca P. Re A. (2015). Detecting modules in biological networks by edge weight clustering and entropy significance. *Front. Genet.* 6:265. 10.3389/fgene.2015.00265 26379697 PMC4551098

[B32] Lee H. Kang S. Kim W. (2016). Drug repositioning for cancer therapy based on large-scale drug-induced transcriptional signatures. *PLoS One* 11:e0150460. 10.1371/journal.pone.0150460 26954019 PMC4783079

[B33] Li C. Luo L. Wei S. Wang X. (2018). Identification of the potential crucial genes in invasive ductal carcinoma using bioinformatics analysis. *Oncotarget* 9 6800–6813. 10.18632/oncotarget.23239 29467930 PMC5805516

[B34] Li J. Li F. Wang H. Wang X. Jiang Y. Li D. (2012). Wortmannin reduces metastasis and angiogenesis of human breast cancer cells via nuclear factor-κB-dependent matrix metalloproteinase-9 and interleukin-8 pathways. *J. Int. Med. Res.* 40 867–876. 10.1177/147323001204000305 22906259

[B35] Linklater E. S. Tovar E. A. Essenburg C. J. Turner L. Madaj Z. Winn M. E. (2016). Targeting MET and EGFR crosstalk signaling in triple-negative breast cancers. *Oncotarget* 7 69903–69915. 10.18632/oncotarget.12065 27655711 PMC5342523

[B36] Liu Y. Yin X. Zhong J. Guan N. Luo Z. Min L. (2017). Systematic identification and assessment of therapeutic targets for breast cancer based on genome-wide RNA interference transcriptomes. *Genes* 8:E86. 10.3390/genes8030086 28245581 PMC5368690

[B37] Lopes C. T. Franz M. Kazi F. Donaldson S. L. Morris Q. Bader G. D. (2011). Cytoscape web: an interactive web-based network browser. *Bioinformatics* 26 2347–2348. 10.1093/bioinformatics/btq430 20656902 PMC2935447

[B38] Maiti A. Takabe K. Hait N. C. (2017). Metastatic triple-negative breast cancer is dependent on SphKs/S1P signaling for growth and survival. *Cell. Signal.* 32 85–92. 10.1016/j.cellsig.2017.01.021 28108260 PMC5731460

[B39] Mardinoglu A. Boren J. Smith U. Uhlen M. Nielsen J. (2018). Systems biology in hepatology: approaches and applications. *Nat. Rev. Gastroenterol. Hepatol.* 15 365–377. 10.1038/s41575-018-0007-8 29686404

[B40] Mardinoglu A. Nielsen J. (2015). New paradigms for metabolic modeling of human cells. *Curr. Opin. Biotechnol.* 34 91–97. 10.1016/j.copbio.2014.12.013 25559199

[B41] Mertins P. Mani D. R. Ruggles K. V. Gillette M. A. Clauser K. R. Wang P. (2016). Proteogenomics connects somatic mutations to signalling in breast cancer. *Nature* 534 55–62. 10.1038/nature18003 27251275 PMC5102256

[B42] Murphy C. G. Dickler M. N. (2015). The role of CDK4/6 inhibition in breast cancer. *Oncologist* 20 483–490. 10.1634/theoncologist.2014-0443 25876993 PMC4425391

[B43] Nagahashi M. Tsuchida J. Moro K. Hasegawa M. Tatsuda K. Woelfel I. A. (2016). High levels of sphingolipids in human breast cancer. *J. Surg. Res.* 204 435–444. 10.1016/j.jss.2016.05.022 27565080 PMC5002890

[B44] Nuncia-Cantarero M. Martinez-Canales S. Andrés-Pretel F. Santpere G. Ocaña A. Galan-Moya E. M. (2018). Functional transcriptomic annotation and protein–protein interaction network analysis identify NEK 2, BIRC5, and TOP2A as potential targets in obese patients with luminal a breast cancer. *Breast Cancer Res. Treat.* 168 613–623. 10.1007/s10549-017-4652-3 29330624 PMC5842257

[B45] Ocana A. Pandiella A. (2015). Targeting oncogenic vulnerabilities in triple negative breast cancer: biological bases and ongoing clinical studies. *Oncotarget* 8 22218–22234. 10.18632/oncotarget.14731 28108739 PMC5400659

[B46] Orchard S. Ammari M. Aranda B. Breuza L. Briganti L. Broackes-Carter F. (2014). The MIntAct project - intAct as a common curation platform for 11 molecular interaction databases. *Nucleic Acids Res.* 42 D358–D363. 10.1093/nar/gkt1115 24234451 PMC3965093

[B47] Perou C. M. Sørlie T. Eisen M. B. van de Rijn M. Jeffrey S. S. Rees C. (2000). Molecular portraits of human breast tumours. *Nature* 406 747–752. 10.1038/35021093 10963602

[B48] Persico M. Ceol A. Gavrila C. Hoffman R. Florio A. Cesareni G. (2005). HomoMINT: An inferred human network based on orthodology mapping of protein interactions discovered in model organisms. *BMC Bioinformatics.* 6(Suppl. 1):S21. 10.1186/1471-2105-6-S1-S21 16351748 PMC1866386

[B49] Prasad T. S. K. Kandasamy K. Pandey A. (2009). Human protein reference database and human proteinpedia as discovery tools for systems biology. *Methods Mol. Biol.* 577 67–79. 10.1007/978-1-60761-232-2_6 19718509

[B50] Purwaha P. Gu F. Piyarathna D. W. B. Rajendiran T. Ravindran A. Omilian A. R. (2018). Unbiased lipidomic profiling of triple-negative breast cancer tissues reveals the association of sphingomyelin levels with patient disease-free survival. *Metabolites* 8 1–14. 10.3390/metabo8030041 30011843 PMC6161031

[B51] Rietman E. A. Platig J. Tuszynski J. A. Lakka Klement G. (2016). Thermodynamic measures of cancer: gibbs free energy and entropy of protein–protein interactions. *J. Biol. Phys.* 42 339–350. 10.1007/s10867-016-9410-y 27012959 PMC4942417

[B52] Salwinski L. (2004). The database of interacting proteins: 2004 update. *Nucleic Acids Res.* 31 248–250. 10.1093/nar/gkh086 14681454 PMC308820

[B53] Schaefer M. H. Fontaine J. F. Vinayagam A. Porras P. Wanker E. E. Andrade-Navarro M. A. (2012). Hippie: integrating protein interaction networks with experiment based quality scores. *PLoS One* 7:e31826. 10.1371/journal.pone.0031826 22348130 PMC3279424

[B54] Siegel R. L. Miller K. D. Jemal A. (2019). Cancer statistics, 2019. *CA. Cancer J. Clin.* 69 7–34. 10.3322/caac.21551 30620402

[B55] Solzak J. P. Atale R. V. Hancock B. A. Sinn A. L. Pollok K. E. Jones D. R. (2017). Dual PI3K and Wnt pathway inhibition is a synergistic combination against triple negative breast cancer. *NPJ Breast Cancer* 3:17. 10.1038/s41523-017-0016-8 28649657 PMC5460220

[B56] Strott C. A. Higashi Y. (2003). Cholesterol sulfate in human physiology. *J. Lipid Res.* 44 1268–1278. 10.1194/jlr.R300005-JLR200 12730293

[B57] Tang Y. Li M. Wang J. Pan Y. Wu F. X. (2015). CytoNCA: A cytoscape plugin for centrality analysis and evaluation of protein interaction networks. *BioSystems* 127 67–72. 10.1016/j.biosystems.2014.11.005 25451770

[B58] Teschendorff A. E. Banerji C. R. S. Severini S. Kuehn R. Sollich P. (2015). Increased signaling entropy in cancer requires the scale-free property of protein interaction networks. *Sci. Rep.* 5:9646. 10.1038/srep09646 25919796 PMC4412078

[B59] Turanli B. Grøtli M. Boren J. Nielsen J. Uhlen M. Arga K. Y. (2018). Drug repositioning for effective prostate cancer treatment. *Front. Physiol.* 9:500 10.3389/fphys.2018.00500 PMC5962745 29867548

[B60] Turanli B. Gulfidan G. Arga K. Y. (2017). Transcriptomic-guided drug repositioning supported by a new bioinformatics search tool: geneXpharma. *Omi. A J. Integr. Biol.* 21 584–591. 10.1089/omi.2017.0127 29049014

[B61] Tusher V. G. Tibshirani R. Chu G. (2001). Significance analysis of microarrays applied to the ionizing radiation response. *Proc. Natl. Acad. Sci. U.S.A.* 98 10869–10874. 10.1073/pnas.091062498 11309499 PMC33173

[B62] Ueno N. T. Zhang D. (2011). Targeting EGFR in triple negative breast cancer. *J. Cancer* 2 324–328. 10.7150/jca.2.324 21716849 PMC3119395

[B63] Uhlen M. Zhang C. Lee S. Sjöstedt E. Fagerberg L. Bidkhori G. (2017). A pathology atlas of the human cancer transcriptome. *Science* 80:357. 10.1126/science.aan2507 28818916

[B64] Varadan V. Anastassiou D. (2006). Inference of disease-related molecular logic from systems-based microarray analysis. *PLoS Comput. Biol.* 2:e68. 10.1371/journal.pcbi.0020068 16789819 PMC1479089

[B65] Vitali F. Cohen L. D. Demartini A. Amato A. Eterno V. Zambelli A. (2016). A network-based data integration approach to support drug repurposing and multi-Target therapies in triple negative breast cancer. *PLoS One* 11:e0162407. 10.1371/journal.pone.0162407 27632168 PMC5025072

[B66] Warm M. Kates R. Große-Onnebrink E. M. Stoff-Khalili M. Hoopmann M. Mallmann P. (2010). Impact of tumor biology, particularly triple-negative status, on response to pre-operative sequential, dose-dense epirubicin, cyclophosphamide followed by docetaxel in breast cancer. *Anticancer Res.* 30 4251–4259. 21036749

[B67] Weigman V. J. Chao H.-H. Shabalin A. A. He X. Parker J. S. Nordgard S. H. (2012). Basal-like Breast cancer DNA copy number losses identify genes involved in genomic instability, response to therapy, and patient survival. *Breast Cancer Res. Treat.* 133 865–880. 10.1007/s10549-011-1846-y 22048815 PMC3387500

[B68] West J. Bianconi G. Severini S. Teschendorff A. E. (2012). Differential network entropy reveals cancer system hallmarks. *Sci. Rep.* 2:802. 10.1038/srep00802 23150773 PMC3496163

[B69] Xiong J. Liu J. Rayner S. Li Y. Chen S. (2010). Protein-protein interaction reveals synergistic discrimination of cancer phenotype. *Cancer Inform* 9 61–66. 20458363 10.4137/cin.s3899 PMC2865773

[B70] Zhang P. Yang X. Yin Q. Yi J. Shen W. Zhao L. (2016). Inhibition of SK4 potassium channels suppresses cell proliferation, migration and the epithelial-mesenchymal transition in triple-negative breast cancer cells. *PLoS One* 11:e0154471. 10.1371/journal.pone.0154471 27124117 PMC4849628

[B71] Zhang Y. G. Du Q. Fang W. G. Jin M. L. Tian X. X. (2008). Tyrphostin AG1478 supresses proliferation and invasion of human breast cancer cells. *Int. J. Oncol.* 33 595–602. 10.3892/ijo_00000045 18695891

[B72] Zhou Y. Lin S. Tseng K. F. Han K. Wang Y. Gan Z. H. (2016). Selumetinib suppresses cell proliferation, migration and trigger apoptosis, G1 arrest in triple-negative breast cancer cells. *BMC Cancer* 16:818. 10.1186/s12885-016-2773-4 27769200 PMC5073736

