# Supplementary figures and images for: Multi-Omic Data Interpretation to Repurpose Subtype Specific Drug Candidates for Breast Cancer
Source: Front Genet. 2019 May 7;10:420. doi: 10.3389/fgene.2019.00420 (PMC6514249; doi:10.3389/fgene.2019.00420)

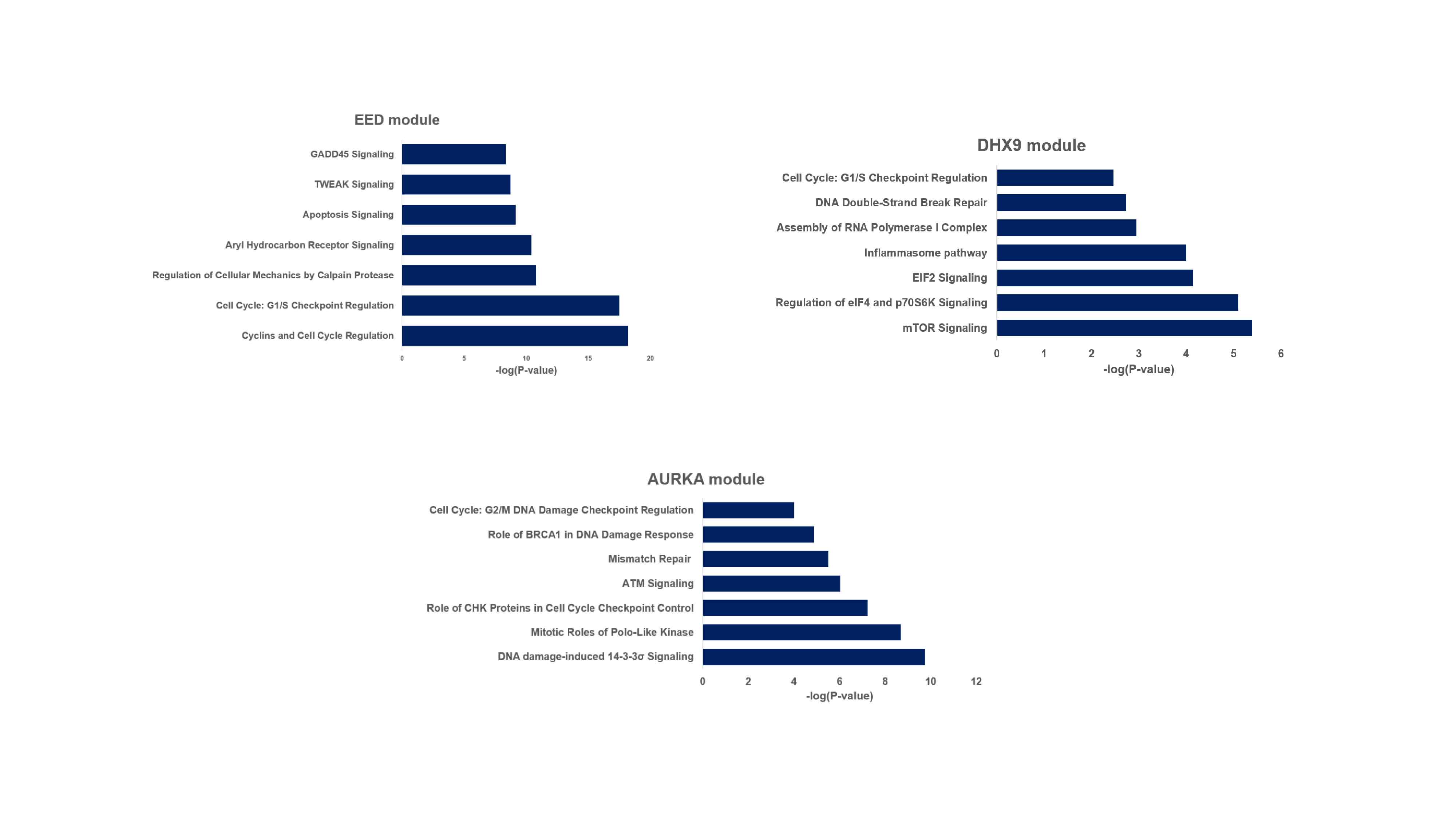

Supplement: FIGURE S1 — Functional enrichment results of the genes involved in each basal-like module using Ingenuity Pathway Analysis (IPA). [file Image_1.JPEG]

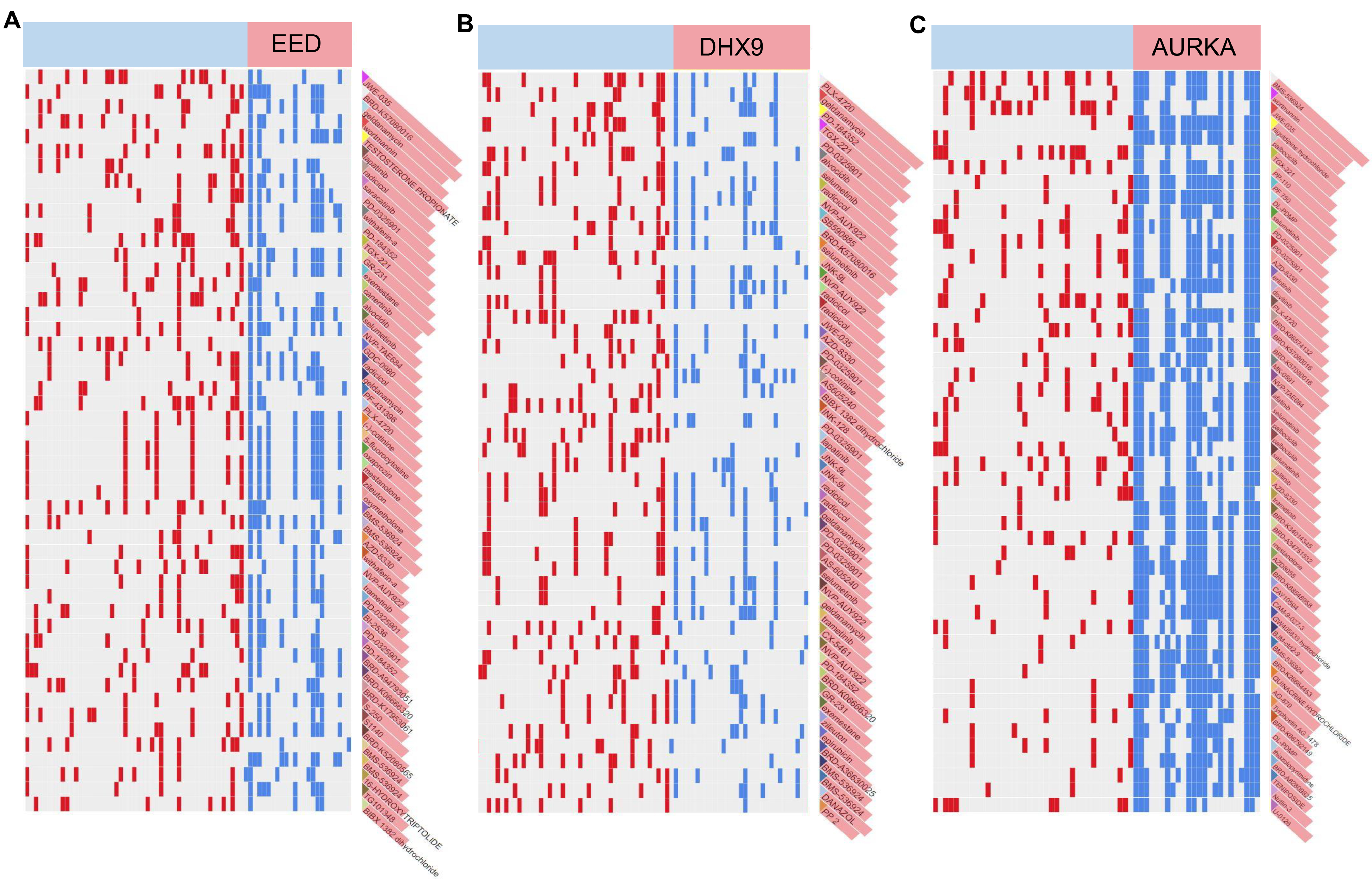

Supplement: FIGURE S2 — The gene signatures of three modules separately on L1000CDS2 for elucidating the differences and similarities between drug-induced expression profiles and disease expression. Drugs were ranked for each module and we elected drugs that showed negatively correlated action mechanisms with the module gene signatures to reverse disease gene expression. [file Image_2.JPEG]
